# Tumeur stromale grêlique associée à la maladie de Von Recklinghausen

**DOI:** 10.11604/pamj.2014.18.160.3559

**Published:** 2014-06-18

**Authors:** Karim Ibn Majdoub Hassani, Younes Aggouri

**Affiliations:** 1 Faculté de Médecine et de Pharmacie de Fès, Université Sidi Mohammed Ben Abdellah, Département de Chirurgie, CHU Hassan II, Fès, Maroc

**Keywords:** Maladie de Von Recklinghausen, tumeur, intestin grêle, Von Recklinghausen disease, tumor, small intestine

## Image en medicine

Les tumeurs conjonctives gastro-intestinales (GISTs) associées à la maladie de Von Recklinghausen (VRH) doivent être analysées en faveur du nouveau concept des GISTs qui se base sur les apports modernes de l'immunohistochimie. Nous rapportons un cas d'un malade présentant l'association d'une tumeur stromale du grêle à la maladie de VRH. Il s'agit d'un patient de 33 ans, qui présente une semaine avant son admission des douleurs abdominales associées à un syndrome subocclusif. L'examen général retrouve une ptose palpébrale droite (A), des tâches café au lait diffuses à tout le corps avec lentigines et neurofibromes pléxiformes et nodulaires (B). On note à l'examen abdominal une distension avec sensibilité diffuse. Une TDM réalisée est en faveur d'une GIST (C). L'exploration chirurgicale note la présence d'une petite formation pédiculée de 4cm de diamètre sur la paroi externe du grêle à 2.5 m de l'angle de Treitz (D). Une résection grêlique emportant la tumeur est réalisée. L’étude histologique est en faveur de GIST. Le diagnostic d'une maladie de VRH est retenu devant les constats dermatologiques et l'atteinte familiale. Au cours de la maladie de VRH, la présence d'une GIST survient bien après les lésions cutanées et son traitement repose sur la chirurgie dont la seule limite reste le caractère multiple des localisations.

**Figure 1:**
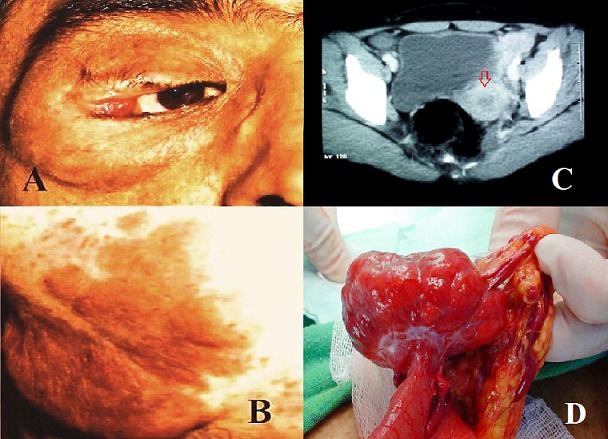
(A): ptose palpébrale droite. (B): tâches café au lait. (C): TDM abdominale montrant une image en faveur d'une GIST grêlique. (D): image per opératoire montrant la tumeur stromale grêlique

